# Radiographers’ conceptualisation of trauma imaging in Gauteng, South Africa

**DOI:** 10.4102/hsag.v29i0.2526

**Published:** 2024-02-29

**Authors:** Shabnam Wahid, Shantel Lewis, Yasmin Casmod

**Affiliations:** 1 Department of Medical Imaging and Radiation Sciences, Faculty of Health Science, University of Johannesburg, Johannesburg, South Africa

**Keywords:** trauma, trauma imaging, radiographers, conceptualisation, medical imaging, experiences, Diagnostic radiographer

## Abstract

**Background:**

Radiographers form part of the healthcare team and are integral in the diagnosis and treatment of trauma patients. Additionally, they are required to provide their services to multiple departments within the hospital, including the emergency department. Healthcare workers who work with trauma patients experience changes in their psychological functioning. Therefore, diagnostic radiographers may have similar experiences; however, limited studies were found on radiographers’ conceptualisation of trauma imaging.

**Aim:**

The aim of this study was to explore and describe radiographers’ conceptualisation of trauma imaging.

**Setting:**

One-on-one in-depth interviews were conducted virtually with radiographers in both the private and public healthcare sectors in Gauteng, South Africa.

**Method:**

In this qualitative, explorative and descriptive study, 20 radiographers were interviewed virtually through Zoom or WhatsApp video calls or telephone interviews. Participants were asked a central question: ‘What does trauma imaging mean to you?’ Detailed notes were taken during the interviews, and interviews were audio-recorded. The data was transcribed and underwent thematic analysis. Trustworthiness and ethical principles were adhered to throughout the study.

**Results:**

Thematic analysis identified four themes: (1) COVID-19 pandemic; (2) road accidents; (3) gender-based violence (GBV); and (4) paediatric injuries that participants conceptualised as trauma imaging.

**Conclusion:**

Participants conceptualised trauma imaging as COVID-19, road accidents, GBV and paediatric patients. It was noted that participants’ personal experiences were significant contributors to their meaning-making and responses to trauma imaging.

**Contribution:**

The study has contributed to the understanding of the conceptualisation of trauma imaging from the perspective of diagnostic radiographers.

## Introduction

Trauma has been extensively studied and debated since the emergence of psychoanalysis in the late 19th century (Cypress 2020:397). ‘Trauma’ is derived from the ancient Greek word ‘*traumatizo*’, meaning wound, physical wound or a bodily injury caused by an external force (Reuther 2012:437). Furthermore, Sigmund Freud added trauma as being a ‘wound of the mind’, and was later adapted and referred to as ‘a psychobiological wound’ (Kostova 2013:68). Sigmund Freud theorised, trauma as an instance of excessive stimulation leading to anxiety that the psyche cannot control, tends to overpower the ego, and leads to a feeling of helplessness (Cypress 2020:397–398). Traumatic events can be isolated or be part of a continuous pattern of incidences, and the perception of the traumatic event is determined by the individual’s personal history and subjective interpretation (Cypress 2020:398).

Traumatic injuries, including burns, motor vehicle accidents (MVAs), violence and assaults, account for 8% of global mortality (WHO 2021b). The global status report on road safety initiated by the World Health Organization (WHO 2018) states that 1.35 million people die annually because of MVAs, and a further 20–50 million people suffer non-fatal injuries. The Road Safety Annual Report (2019) in South Africa states that the long-term trend for road fatalities has been on an upward trend since 2006. Fatal MVAs on South African roads increased from 8405 in 2020 to 10611 in 2021 (State of Road Safety Report 2021). The MVA fatality increase in Gauteng over the 2022/2023 festive period is concerning after being compared to that of the previous year (Mbalula 2023). In addition to these statistics, it is estimated that dozens of casualty patients are hospitalised for every traumatic death that occurs (WHO 2021a).

South Africa has the third-highest crime rate globally, which may be attributed to poverty, socio-economic disparities, poor service delivery, and an inadequate criminal justice system, among other factors (World Population Review 2021).

Medical management of trauma patients requires speed, accuracy and efficiency within the first 60 min of the trauma patient arriving at the emergency department to ensure improvement of the patient’s condition; known as the ‘golden hour’ (Hayre & Cox 2020:163; Raja & Zane 2021). Medical imaging is essential in this golden hour (Raja & Zane 2021). Medical imaging forms an integral part of the diagnosis and treatment of a wide range of injuries and illnesses, and radiographers have the responsibility to provide radiographic images by integrating patient history, clinical data, and imaging procedures (Health Professions Council of South Africa [HPCSA] 2020:2). The radiographer is essential in providing medical imaging of trauma patients, and their immediate response in a highly pressurised environment requires radiographers to expeditiously obtain high-quality images, while ensuring radiation safety measures for the patient and the entire healthcare team (Hayre & Cox 2020:164). While trauma radiographic projections and positioning are stated in radiographic textbooks (Lampignano & Kendrick 2020), Cypress’s (2020:398), notion of trauma being determined by the individual’s personal history and subjective interpretation remains unexplored. In this regard, there is no current literature on how radiographers understand trauma imaging and what trauma imaging means to them. Therefore, this study was undertaken to explore and describe radiographers’ conceptualisation of trauma imaging. Conceptualisation refers to the breaking and conversion of ideas into common meanings to develop an understanding of the idea within a specific context (Sequeira 2014). In this study, the conceptualisation is understanding trauma imaging from a radiographer’s context.

Gauteng was selected as the setting for this study because it is the smallest but the most densely populated of the nine provinces in South Africa and reported the highest number of contact crimes compared with the other provinces in South Africa between April and June 2023 (South African Police Service [SAPS] 2023:14). Adding to the crime rate, Gauteng also reported high rates of MVAs (Mbalula 2023). The high crime and accident rates proportionally impact the volume of violent and traumatic cases requiring medical management and admission to hospitals (Hardcastle et al. 2016:180). As a result of violence, a high number of patients seek emergency medical treatment. Therefore, it could be surmised that radiographers working in Gauteng would experience imaging trauma patients more frequently (Creswell & Creswel, 2018:257).

## Research methods and design

### Study design

A qualitative, explorative and descriptive approach was selected to explore and describe radiographers’ conceptualisation of trauma imaging (Green & Thorogood 2018:19). Qualitative studies are used to determine and gain an understanding of events by looking at people’s experiences, beliefs and views within their own environment (Green & Thorogood 2018:7). A central focus of qualitative research is to observe and describe a specific phenomenon from the subjective realities of the research participants (Creswell & Creswell 2018:50). Therefore, a qualitative explorative and descriptive design was selected to understand how radiographers conceptualise trauma imaging.

### Setting

The study was set in the Gauteng province. Radiographers may work in private and public hospitals in South Africa. In public hospitals, the radiology department falls within the purview of hospital management, and in private hospitals, radiology departments are privately owned practices that are managed independently from the hospital. Radiology departments in both public and private hospitals were included in this study.

### Study population and sampling strategy

The population were all diagnostic radiographers registered with the HPCSA over the age of 18 years and working in both private and public healthcare settings in Gauteng. Radiology departments in Gauteng were contacted telephonically to enquire if trauma patients were imaged daily at the respective hospitals. From the responses, five trauma hospitals representing both the public and private sectors were purposively sampled based on the influx of trauma patients at these hospitals (Green & Thorogood 2018:75). In addition, these five trauma hospitals were selected as written consent was obtained to conduct the study from the practice managers and/or chief executive officers (CEO) at the respective practices. Two hospitals were level-one accredited trauma centres in Gauteng, South Africa. This certification approves the accredited centres to treat all levels of trauma injuries, including critical care patients presenting with multiple injuries (Netcare Hospitals 2020).

### Data collection

The data process began once approval and ethical clearance of the study was obtained from the Faculty of Health Science Higher Degrees Committee and the Research Ethics Committee at the University of Johannesburg. The private hospitals were contacted by phone to request permission to conduct the research study at the practice. The study information letter was then emailed to the department heads of the private practices, who agreed. For hospitals in the public sector, Gauteng Health Department approval was applied online through the National Health Research Database. The assistant directors of the radiology departments and/or CEOs of the public hospitals were then approached for permission to conduct the study. Once permissions were obtained, the researcher physically visited the five radiology departments to introduce the radiographers to the study. All radiographers at these practices were invited to participate in the study during a group session held by the researcher and the head of the department. Information letters and consent forms were then distributed and interested radiographers approached the researcher after the group session to share contact details. These radiographers were then contacted individually, and a date and time were arranged to conduct one-on-one interviews. Participants needed to sign consent to participate in the study and for the audio recording of interviews prior to the interview.

Data collection occurred between June and August 2020. Because of lockdown restrictions in South Africa as a result of the COVID-19 pandemic, 9 in-depth interviews were conducted virtually through Zoom or WhatsApp Video, and 11 interviews were conducted telephonically as per the participants’ request. Verbal consent for the interview and the audio recording were obtained at the beginning of each interview, and all interviews were recorded using an Olympus WS-853 Digital Stereo Voice Recorder. All interviews were conducted at times that were convenient for the research participants as they needed to feel safe enough to openly discuss their experiences (Green & Thorogood 2018:149). A single central question was posed that allowed the participants to express their feelings: ‘What does trauma imaging mean to you?’ Participants’ responses guided follow-up questions (Creswell & Creswell 2018:257). Follow-up questions included: ‘Can you explain further?’ ‘How long ago?’ ‘Were you alone?’ ‘Can you share an example?’

Because of the study being qualitative in nature, comprehensive notes were taken on additional information such as body language, expressions and additional non-verbal communication that were of value during the interviews (Creswell & Creswell 2018:263). This was only possible for interviews conducted via Zoom or WhatsApp Video. Notes were taken detailing any changes in tone during the telephonic interviews.

Because of the nature of the study, participants could have potentially unearthed a traumatic experience. For this reason, a trauma counsellor was on standby to offer psychological aid to participants who may have needed it. The trauma counsellor was not present while interviews were being conducted to ensure the research participants’ confidentiality. If participants felt that they required the trauma counsellor’s services, they were encouraged to notify the researcher; however, none of the participants requested this service. The researcher met with the supervisors to reflect on the psychological effect of the interviews, debrief and discuss the interviews. Interviews continued until data saturation was reached, yielding 20 interviews (Green & Thorogood 2018:77). Prior to data collection, the researcher started a journal to apply bracketing and continued journalling to ensure reflexivity and limit bias.

### Data analysis

Data analysis and collection occurred simultaneously in this study (Creswell & Creswell 2018:306). During the analysis process, bracketing was applied to exclude the researcher’s feelings and opinions. Braun and Clarke’s (2021) method of thematic analysis was used in this study. The six-step process involved data familiarisation, coding, initial theme generation, reviewing themes, defining and naming of themes, and writing up the themes in the report (Braun & Clarke 2021:4). The data analysis process began with the verbatim transcription of the audio-recorded interviews. Transcribed interviews were read and reread for further familiarisation with the information (Green & Thorogood 2018:259).

Twenty transcribed interviews were then analysed, and informal notes were made, beginning the manual coding process. Field notes were also used in conjunction with the transcriptions to develop codes (Creswell & Creswell 2018:269). Themes were generated by identifying and organising the codes into patterns. The transcriptions were also sent to an independent coder, and a consensus meeting was held to finalise the themes. The themes needed to accurately represent participants’ experiences; therefore, an inductive method of analysis was used to generate these themes (Creswell & Creswell 2018:272). Four themes were identified, named and defined. Themes and categories were supported with verbatim quotations from the participants.

### Trustworthiness

Lincoln and Guba’s measures of trustworthiness (1985:296–330) – credibility, dependability, transferability and confirmability, were used to ensure the trustworthiness of the study. Credibility in this study was achieved through prolonged engagement, reflexivity and triangulation of interview data with the researcher’s notes and literature as a way to provide multiple perspectives (Creswell & Creswell 2018:315). Dependability was ensured by keeping an audit trail and by providing a detailed description of data gathering, analysis and interpretation. Transferability was ensured by describing the research setting in detail to allow for comparison to a different setting (Green & Thorogood 2018:309). Confirmability mirrors replicability and accuracy in the description of data, and results can be confirmed by referring to the audit trail (Creswell & Creswell 2018:275). Bracketing, reflexivity and member checking were used to ensure that the results were confirmable and unbiased.

### Ethical consideration

Permission to conduct this study was sought from the University of Johannesburg Faculty of Health Sciences Research Ethics Committee (REC-184-2019). Additional permission was sought from the practice managers of private radiology departments and the Gauteng Health Department through the National Health Research Database for radiology departments in the public sector. Participants in this study were treated with respect and dignity throughout the research process.

Radiographers at the selected hospitals were provided with a study information letter explaining what the research entailed and their involvement as research participants. Written and verbal consent was obtained from the participants for the one-on-one interviews and the audio recordings prior to any data collection. Research participants and institution sites were deidentified to ensure their privacy, and confidentiality rights were respected. Interviews were coded first by the researcher and then by an independent coder. The coder was required to sign a confidentiality agreement respecting the participants’ right to privacy and confidentiality. While there was no risk of any physical harm to the participants because of the nature of the study, an anticipated risk was the possibility that participants could unearth a traumatic experience. A trauma counsellor was on standby should a participant require this service.

## Results and discussion

Twenty radiographers participated in the study, 10 from private hospitals and the remaining from public hospitals. Participants ranged from 25 years to 64 years, and had an average of approximately 6 years of experience in trauma imaging. Two participants were over the age of 50, one of whom left the profession and then returned. The participants’ demographics are outlined in Table 1.

**TABLE 1:** Demographics of participants.

Participant	Age (in years)	Private or public hospital	Experience in trauma imaging (in years)
1	29	Private	7
2	29	Private	6
3	31	Private	7
4	64	Private	12
5	33	Private	6
6	27	Public	4
7	26	Public	2
8	28	Public	5
9	27	Private	3
10	27	Public	5
11	57	Private	10
12	27	Public	4
13	30	Public	9
14	26	Private	2
15	25	Public	2
16	32	Public	10
17	36	Public	7
18	27	Private	3
19	37	Private	9
20	33	Public	8

It became apparent that when participants were asked the central question: ‘What does trauma imaging mean to you?’ each participant had a different interpretation of what trauma imaging meant to them. It was noticed that participants’ personal experiences were significant contributors to their responses when imaging trauma patients. Thematic analysis identified four themes: (1) COVID-19 pandemic, (2) road accidents, (3) gender-based violence (GBV), and (4) paediatric injuries that participants conceptualised as trauma imaging.

### Theme 1: COVID-19

Participants highlighted the type of trauma patients they imaged and which cases were traumatic. Data were collected in 2020 when there was much uncertainty around COVID-19; therefore, participants’ concept of trauma imaging included COVID-19. Participants felt restricted in displaying compassion and care towards their patients because of the personal protective equipment (PPE) that all healthcare workers treating COVID-19-infected patients were required to wear. Forms of non-verbal communication, such as a smile, often went unseen as the use of face masks was compulsory. Restrictions placed on physical contact between the participants and their patients resulted in heightened empathy for the infected:

‘In this COVID time now, it is difficult for me to empathise with my patients, because I am the type of person where I see you need a hug, I will hug you…which I can’t do, and I find myself apologising to my patients to say I am sorry I cannot hug you now ….’ (P2, female, 29 years old)

‘You cannot pat her back. You cannot rub her hand. You are not allowed to touch. You cannot smile. You cannot show any like affection. You are not allowed to actually be close to this patient. So that really hit me, because the reason why I went into radiography is to help people and tell them listen, it will be okay. So now you need to like treat this patient as this big virus and be so inhumane.’ (P9, female, 27 years old)

Participants expressed that they not only had to deal with the pandemic at work, but were also afraid to transmit the virus to their families at home:

‘You cannot exactly stay away from your family as much when you live with them. It is not as easy as it should be or it sounds, you know? You cannot isolate yourself from the people you live with … So I always worry about [name withheld]. Like what if I had to take it home to him and stuff like that.’ (P3, female, 31 years old)

‘It becomes traumatic, especially when you get people that are close to you testing positive.’ (P8, female, 28 years old)

Globally, the world population was not prepared for the COVID-19 pandemic. Because of the rapid transmission rate of the COVID-19 virus, many countries around the world implemented lockdowns to reduce the number of lives lost to this virus (Greyling, Rossouw & Adhikari 2021:1). South African level five lockdown regulations remained some of the strictest globally (Smart, Broadbent & Combrink 2020). Residents were restricted from leaving their homes except to purchase groceries and medical care. Social gatherings were banned, strict curfews were imposed, and all non-essential stores and services were shut down. Together with the alcohol ban, these regulations resulted in a significant reduction in road accidents and, as a result, hospital admissions. Although the pandemic resulted in a decrease in trauma cases all over the world, several participants discussed the pandemic as part of their trauma experience (Giudici et al. 2021:7).

As COVID-19 complications include respiratory conditions, routine chest imaging is required to monitor the progression of the disease (Elshami et al. 2020:1). As a result, radiographers are part of the frontline healthcare team in the fight against the COVID-19 pandemic. Therefore, all the participants were directly involved in imaging COVID-19 patients at their respective hospitals. The uncertainty accompanying this worldwide pandemic resulted in high levels of stress and frustration experienced by the participants. Participants shared their fear of infecting friends and family at home with the disease, which is consistent with the findings of a Gauteng study on diagnostic radiographers’ experiences of COVID-19 (Lewis 2023:81). Multiple studies show a similar impact of COVID-19 on healthcare workers (Van de Venter et al. 2021:586–594).

### Theme 2: Road accidents

Participants expressed that they considered road accidents as traumatic. The participants easily recalled experiences and specific details that occurred several years ago. Although healthcare workers are aware of limb amputations as a result of severe injury, participants of this study still displayed distress and a feeling of sympathy for road accident patients who had limb amputations. Additional sadness and despair were experienced, knowing that the patients’ lives would never be the same:

‘It was a 28-year-old male that had been run over by a truck and I was doing a Lodox for him … I knew they were going to amputate, but it was still a shock to hear that they amputated one leg above the knee and one leg below the knee and I kept thinking to myself this man is going to walk around this hospital with no legs.’ (P6, female, 27 years old)

‘… It was a train accident. The guy was drunk. He tried to commit suicide. Both his legs were off. He had a tension pneumo. One humerus was just hanging by a piece of the skin, you know? It was just held together by the skin. He also had a huge fracture in the skull as well, you know?.’ (P8, female, 28 years old)

Another participant detailed a graphic story of imaging a patient’s skull in the emergency department without knowing that the patient had a severe open skull wound. This incident resulted in the participant’s hand inside the patient’s skull. Lasting feelings of regret overwhelmed this participant as the patient passed away:

‘it was an MVA patient, but the patient had a huge skull fracture and at the time we did not have stitches so I had to do the skull x-ray, but when I tried to lift the patient’s head to do the skull x-ray, my hands went into the patient’s skull.’ [sic] (P8, female, 28 years old)

Participants expressed feelings of being the indirect cause of their patients’ deaths. Although participants light-heartedly mentioned other healthcare workers commenting on patients dying whenever they were present for imaging, this clearly affected them psychologically:

‘… a lot of the patients that I was going to x-ray had just passed away … and that was psychologically affecting me’ (P12, female, 27 years old)

‘I have had quite a lot of patients who have crashed while I was busy with them. So much so that it got to a point where if I had to go to resus and there is a critical patient, they would make a joke and say no, don’t do that patient. Please call somebody else, you know?.’ (P8, female, 28 years old)

As a result of road accidents, patients present with severe injuries requiring medical attention. Patients with severe injuries should be treated at higher-level trauma hospitals to improve their chances of survival (Hardcastle et al. 2016:181). Participants in this study worked in private and public sectors, and therefore formed part of the medical management of the patient. According to the WHO (2021a), more than 90% of road accident deaths occur in low- to middle-income countries (LMICs), and death rates are highest in the African region. Common admissions to the emergency department include MVAs, pedestrian-vehicle accidents, and motorbike accidents. On a typical weekend in South Africa, the Emergency Medical Services ER24 contact centre typically receives between 200 and 350 road emergencies between Friday and Sunday (De Wet 2020). Injuries are traumatic not only for the patients who are directly experiencing physical trauma, but possibly also for the healthcare workers who treat the injuries (Shoji et al. 2015:2). Participants’ narratives included vivid details of road accidents that contextualised their traumatic experiences. Medical emergency workers are often faced with situations in which human suffering, visible distress, and pain are witnessed, impacting their mental health (Maia & Ribeiro 2010:296). Observing an injured person struggling to hold on to their life results in emotional strain on first responders (Shoji et al. 2015:2). Similarly, participants in this study experienced distress and emotional strain in imaging patients presenting with severe injuries because of road accidents.

### Theme 3: Gender-based violence

Participants relayed stories of abuse against women and the severe trauma as a result of the abuse. Participants’ reactions to GBV trauma cases included the need to offer aid, assistance, and support to victims:

‘I remember I had a patient one day who was assaulted by her boyfriend and I was x-raying her. She had a new fracture on her humerus, but she also had old injuries. A broken finger that was deformed and she had never had it seen to. So it healed in that displaced position and I remember myself and my colleague were saying you know, you don’t deserve this. You should not be with a man who treats you like this. It just … I suppose we are overstepping, but we just feel we need to give another woman that support … And as we wheeled her out of the room she phoned her boyfriend to say why are you not coming to see me? I am in hospital. You did this to me. Please come and see me. I got so upset with her that I actually shouted at her and I said why would you do that? Why would you call him here if he does not want to be here? He clearly does not care about you and I realised how unprofessional I was being, but I could not help it and I was upset with her for the whole day.’ (P6, female, 27 years old)

Participants found it difficult to detach themselves from GBV patients, often questioning why victims never sought help:

‘the domestic violence patients … I can think about these patients for days on end seeing what they went through … It sticks with me.’ (P6, female, 27 years old)

While dealing with traumatised patients, specifically victims of GBV, care needs to be taken to ensure that the situation is approached with caution and sensitivity. Participant 6 displayed contrasting behaviour. Reasons for this may be attributed to no explicit formal training in dealing with GBV patients, resulting in increased anxiety when faced with victims of GBV and contrasting reactions.

Since the implementation of worldwide lockdowns to curb the spread of COVID-19, increase in domestic violence cases have been reported globally (Connolly et al. 2020). Gender-based violence has been a constant struggle in South Africa, with women and young girls commonly affected. Healthcare workers play an essential role in the well-being and recovery of GBV victims (Wallin-Lundell et al. 2018:5). Often healthcare workers are the first contact for these victims of GBV, and thus are the first to identify psychological or physical signs of trauma history (Jiménez-Rodríguez et al. 2020:2). Radiographers are often the first to view internal and previous injuries because of the nature of their job, resulting in feelings of anger and helplessness as expressed by participants (Pérez-Tarrés, Espinosa & Pereira da Silva 2018:220). President Cyril Ramaphosa (2021) declared GBV the second pandemic in South Africa. Therefore, because of the high incidence of GBV participants’ stories were congruent.

### Theme 4: Paediatric injuries

Participants recalled paediatric trauma experiences vividly as it emotionally affected them. Often relationships were formed between the participants and their paediatric patients as it was difficult to detach emotionally from abused or injured children:

‘it is like motherly instinct almost that kicks in….There was a little kiddy who came in. She and her dad was actually driving on a motorcycle and they overtook a truck and ja, hit a car and she flew over her dad. So ja. I am actually getting like heart palpitations just thinking about it.’ (P9, female, 27 years old)

Participants offered support and even attempted to counsel paediatric victims as a parent would. When faced with a paediatric trauma case, participants were observed to follow up after imaging to ensure that the proper medical attention was given to the victims:

‘that child had been abused by the stepmom and had healed fractures and new fractures as well. The child was very scared …and she did not want to open up to anybody else except me … and I mean, at that time I was not even thinking of recording this child so that they get information, but with me, with paeds, I think I am more of a counsellor type of person and child loving person. So I prefer to sit with kids and get to understand and I want to know the cause, you know? How it got sore and what did they do to help this child?.’ (P17, female, 36 years old)

A specific trauma case that remained with a participant for approximately 40 years was that of a paediatric burn injury, which resulted in the participant vomiting after having imaged the trauma patient. The participant experienced vivid visual and olfactory memories, which triggered the reliving of the traumatic event:

‘… two kids were playing in a dog kennel, two cousins and they got hold of … matches and I don’t know if it was methylated spirits or petrol and they actually set the kennel alight with them inside it. I was called to do chest x-rays and it literally looked like I was looking at a chicken that had been burnt with the black legs and the arms … he was burnt to a char… this woman who was clearly very drunk, very very drunk, who told me that her kid got burned and that she just carried on partying, but now she wants to check out her kid …. The following day we went down to the river to … have a picnic and I was actually … I was so traumatised that I actually could not speak to anybody. I was totally sort of secular and they were sort of braaing their meat and then I just started vomiting, because that smell of that burnt flesh … and looking back now, I mean this was many years ago. I needed heavy counselling then.’ (P4, female, 64 years old)

Paediatric admissions to the hospital are often daunting for inexperienced staff members (Meadors et al. 2010:105). Treatment methods vary, and often, children are full of fear, which can create some difficulty for healthcare workers (Karisalmi, Stenhammar & Kaipio 2018:60). Paediatric patients presenting in the emergency departments before the COVID-19 pandemic constituted 80% of trauma cases resulting from road accidents and children not strapped securely by a seatbelt (Mlamla 2019). Other reasons for paediatric patients presenting in the emergency departments are burn injuries and drownings. Contact and liquid burns are generally a result of household items left unattended, such as boiling water and household appliances. Often substance abuse results in parental negligence with fatal injuries to children. Children have a desire to explore; however, if they are left unattended for even a moment, fatal accidents could occur (Cape Times 2020). Drowning is the third most common accidental injury among children under 5 years old in South Africa, after fires and MVAs (Scheepers 2021).

Participants’ narratives included paediatric burns and drowning cases. Non-accidental injuries refer to any injury purposefully inflicted on a child (Weber 2015:1). Witnessing traumatic experiences while offering care to injured patients is known to cause secondary traumatic stress among nursing staff (Günüşen et al. 2019:1). Paediatric Intensive Care Unit patients require more complex treatment methods because of their size and age, which adds to the stress and poses as a challenge for those treating the critically ill (Meadors et al. 2010:105). Similarly, participants in this study expressed difficulty in imaging paediatric patients because of their fragility and compromised health. Because of the highly emotional sensitivity of a paediatric environment, paediatric nurses experience higher levels of stress compared to other nurses (Kellogg et al. 2018:97). They are triggered emotionally by the smell of paediatric burns, the sound of mothers screaming with grief, and even the sight of traumatic injuries (Sheppard 2016:53). This particular finding is consistent with the participant who vomited after the smell of ‘braaied’ meat evoked the memory of the burnt child that she had been required to image.

## Conclusion

This study explored and described Gauteng radiographers’ conceptualisation of trauma imaging. Each participant had a different interpretation of what trauma imaging meant to them. Trauma patients and cases that resonated with the participants were found to have triggered various responses. Participants’ interviews indicated that they found the COVID-19 pandemic, road accidents, GBV, and paediatric injuries to be traumatic. It is recommended that radiographers, their respective clinical practices and higher education institutions review the findings of this study to ensure assistance is provided to support diagnostic radiographers.

## Limitations

One of the major factors that influenced the results of the study was the COVID-19 pandemic. Data collection occurred during the first wave of COVID-19 infections in Gauteng, and as a result of the lockdown regulations, data collection was not face-to-face. Only 9 interviews were conducted through Zoom or WhatsApp Video, and the remaining 11 were conducted telephonically as per the participants’ request. This resulted in some missed non-verbal communication cues. No literature could be found on radiographers’ experiences of trauma imaging presenting a challenge as no comparisons could be made with the existing research findings. Literature was, therefore based on findings from other allied healthcare workers.
